# Planning, Cognitive Reflection, Inter-Temporal Choice, and Risky Choice in Chess Players: An Expertise Approach

**DOI:** 10.3390/jintelligence13030040

**Published:** 2025-03-19

**Authors:** Guillermo Campitelli, Martín Labollita, Merim Bilalić

**Affiliations:** 1 College of Health and Education, Murdoch University, Murdoch, WA 6150, Australia; 2 Facultad de Economia, Universidad de Buenos Aires, Buenos Aires C1428EGA, Argentina; martinlab.eco@gmail.com; 3 Department of Psychology, Northumbria University, Newcastle upon Tyne NE1 8SA, UK; merim.bilalic@northumbria.ac.uk

**Keywords:** expertise, chess, expertise approach, cognitive reflection test, Tower of London, individual differences, transfer

## Abstract

This study investigates the cognitive processes underlying chess expertise by examining planning, cognitive reflection, inter-temporal choice, and risky choice in chess players. The study involves 25 chess players and 25 non-chess players, comparing their performance on the Tower of London (TOL) task, Cognitive Reflection Test (CRT), inter-temporal choice (ITC), and risky choice tasks. Results indicate that chess players outperform non-chess players in TOL and CRT, showing superior planning and cognitive reflection abilities. Chess players also prefer future rewards over immediate ones in ITC, suggesting a higher propensity for future more rewarding options. In risky choice tasks, chess players made more decisions based on expected value than non-chess players, but the evidence in favour of differences between groups is very weak. Despite this study not being able to establish causality, the findings highlight the cognitive advantages associated with chess expertise and suggest potential areas for further research on the transfer of cognitive skills from chess to other domains and differences in general abilities between experts and novices.

## 1. Introduction

Chess has long been considered an ideal domain for investigation of cognition in general and expertise specifically. Chess has been described as the “drosophila” of cognitive science (Blanch 2021; Charness 1992; Gobet et al. 2004; Saariluoma 1995) due to its utility in studying complex cognitive processes in a complex and, yet at the same time, highly controlled environment. Chess as a domain provides a strong ecological validity and the existence of a rating system (Elo 1978) that objectively determines the level of expertise of individuals makes it particularly suitable for empirical investigations (Vaci and Bilalić 2017) and development of psychometric measures (e.g., van der Maas and Wagenmakers 2005).

Because of the importance of chess in cognitive science, researchers have also investigated whether chess players differ from the general population in general cognitive abilities (e.g., De Bruin et al. 2014; Frydman and Lynn 1992; Grabner 2014; Grabner et al. 2007; Waters et al. 2002) and decision-making tasks (e.g., Klein et al. 1995; Sigman et al. 2010; Trincherini and Postal 2024). This research is the starting point for two differing possibilities. If it is found that chess players differ from the general population in general cognitive abilities, it may be the case that differences in general cognitive abilities are important for playing chess, which is important for the field of research aiming to understand individual differences. Alternatively, playing chess may enhance general cognitive abilities, which is important for education and the field of research aiming at understanding the transfer of knowledge or skill acquired in a domain to another domain.

The first line of research has mainly focused on whether chess players differ from the general population in general intelligence or other general abilities. Burgoyne et al. (2016) conducted a meta-analysis and found a small difference in favour of chess players in fluid reasoning (*r* = 0.19) and short-term memory (*r* = 0.25). They also found a moderate effect in mathematical ability (*r* = 0.35) and a small effect in verbal ability (*r* = 0.19) in favour of chess players. This result was consistent with another meta-analysis that reviewed intelligence-related tests (*d* = 0.49; Sala et al. 2017). The second line of research has focused on whether chess training leads to improvements in mathematical abilities, reading abilities, and academic performance, with Sala and Gobet (2016) conducting a meta-analysis that shows again a small effect size of *g* = 0.34 (see also Blanch 2022, for a critical follow up of this meta-analysis and insights on the reasons for small to moderate effect sizes).

We propose that the finding of only small to moderate effect size in previous studies is due to the fact that previous studies mainly focused on measures that do not share common cognitive components with chess. In their model of knowledge transfer, Thorndike and Woodworth (1901; see also Woodworth and Thorndike 1901) indicate that transfer of knowledge or skill from one discipline to another occurs only when the disciplines require people to engage in the same stimulus–response elements or, as proposed in more recent models, the same cognitive elements (Singley and Anderson 1989). There are only a few components that chess and mathematics share, and even less chess and reading ability. Likewise, general intelligence or general memory tests may share some components that are relevant for chess playing but not all of them are relevant.

In this study we aim to capture large population effect sizes by investigating differences between chess players and non-chess players in tasks of a general nature that we believe capture cognitive processes that are shared with playing chess: planning and cognitive reflection. Moreover, given that the preferences for future options and the evaluation of risks in financial options have been found to correlate with cognitive reflection, we investigated differences between chess players and non-chess players in risky choices and intertemporal choices in an exploratory fashion.

### Overview of the Present Study

In this study we compared chess players and non-chess players in four non-domain specific measures broadly categorized as planning, cognitive reflection, intertemporal choice, and risky choice. We now discuss these four categories.

Planning. Chess players engage in a process called look-ahead search (e.g., Charness 1981; Holding 1989, 1992) which allows them to represent future situations ahead from the current position. This process involves: (a) generating candidate moves to be played in a position; (b) sequentially imagining as if those movements occur on the chess board, imagining hypothetical responses of the opponent, imagining responses to these responses and so forth; (c) evaluating the convenience of reaching such positions; and (d) based on the information gathered by this process, making a decision of what move to play in the current position. The seminal works of Adrian De Groot ([1946] 1978) and Herbert Simon and colleagues (e.g., Chase and Simon 1973a, 1973b; Gobet and Simon 1996a, 1996b) laid the foundation for understanding the cognitive mechanisms underlying chess expertise. For example, the template theory of expertise (Gobet and Simon 1996a), an expanded version of the chunking theory (Chase and Simon 1973a, 1973b) indicates that chess expertise is acquired by storing in long-term memory typical configuration of chess pieces (i.e., chunks (3–4 pieces) or templates (larger structure with a core and slots that allow quick addition of information)) linked with typical moves; when a configuration stored in long-term memory appears in a chess position, players apply pattern-recognition processes which lead to the triggering of typical moves. Gobet (1997) (see also Gobet and Simon 1998) indicated that look ahead search is conducted by recursive pattern recognition, which involves mentally simulating the execution of a move and applying pattern recognition on the position reached after the mental execution of that move.

The seminal work of De Groot ([1946] 1978) did not identify differences in depth of search between players of different level of expertise (see also, Bilalić et al. 2008a). This is most likely a consequence of using a single, relatively simple position. Other studies using different positions found that better players search deeper (e.g., Bilalić et al. 2009; Connors et al. 2011; Gobet 1998), with one study (Campitelli and Gobet 2004) finding that a grandmaster could look ahead up to 40 plies (20 white moves and 20 black moves). The evidence indicates that expert’s search process is highly dependent on context. In situations where deep search is required for the successful assessment of the situation, experts tend to search deeper than lesser players (Bilalić et al. 2009).

**Figure 1:**
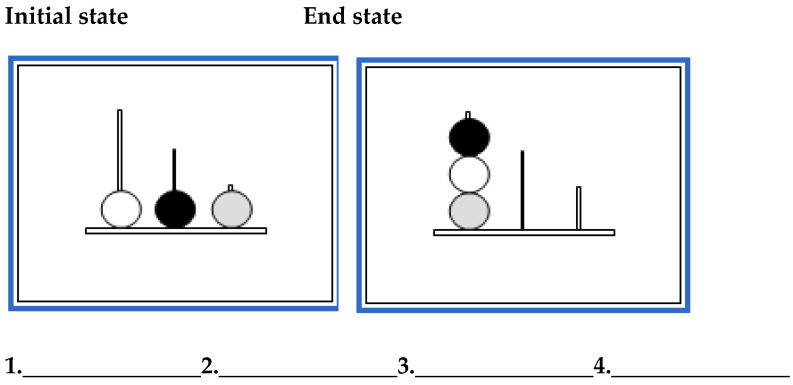
Example of a Tower of London problem.

This thinking ahead in chess resembles the cognitive processes required to solve a so-called planning task: the Tower of London task (TOL, Shallice 1982), which was developed to measure planning deficits in patients with frontal lobe lesions. The TOL task is a standard measure of planning abilities, requiring participants to move balls across pegs to match a target configuration (see Figure 1). As indicated in Unterrainer et al. (2006), Carlin et al. (2000) describes the cognitive processes involved in the Tower of London task as a process that involves mentally generating sequences of moves and evaluating the consequences of each move, maintaining a goal state to which the participants need to achieve, anticipation of future events, and acknowledging when a goal was achieved (see also Kaller et al. 2004). Given the resemblance of look-ahead search in chess and the Tower of London task, we hypothesized that we would find a large effect size when comparing chess players to non-chess players. Two previous studies have investigated this, with Unterrainer et al. (2006) finding that chess players outperformed non-chess players on the Tower of London task, and that this difference was higher in more difficult problems. On the other hand, Unterrainer et al. (2011) included a limit on the time the participants were allowed to think and found no differences between chess players and non-chess players in the same Tower of London task.

Cognitive reflection. Cognitive reflection was defined by Frederick (2005) as “the ability or disposition to resist reporting the response that first comes to mind” (Frederick 2005, p. 35). The cognitive reflection test (CRT) contains three mathematical questions (Pennycook et al. 2016; see Toplak et al. 2014, Thomson and Oppenheimer 2016 for versions of CRT with more questions), which tend to trigger an intuitive response that is not the correct answer. Thus, in order to solve the problem correctly participants need to refrain from providing that answer and engage in (relatively simple) computations to reach the correct answer (see Campitelli and Gerrans 2014 for a mathematical model that captures these component processes of performing the test).

As we introduced earlier, the chunking and template theory indicate that chess players possess chunks and templates which trigger typical moves. Although these moves tend to be good ones, they are not always the best move. Therefore, as in the CRT, it is important for chess players to refrain from making that intuitive move and consider other alternatives. For example, Bilalić et al. (2008b, 2008c) have investigated whether chess players are capable to do this when exposed to a chess position in which the intuitive answer is not the best option. They found that despite the chess players do sometimes choose the intuitive suboptimal answers (Bilalić et al. 2008c; Bilalić and McLeod 2014), they are capable of inhibiting that response and find the best move (Bilalić et al. 2008b). Given that the computational component of the CRT is simple and most people (including chess players) are able to conduct the correct mathematical computation, the essence of the CRT is the inhibition of intuitive answers. Given that this component occurs in chess, we expected chess players to outperform non-chess players in the CRT.

Intertemporal choice. Intertemporal choice tasks do not have a correct answer; rather, they reveal the people’s preferences (Frederick et al. 2002; Keidel et al. 2021; Loewenstein 1992). For example, participants are asked to choose whether they prefer to receive $1000 now or $1100 next month. Given that chess playing involves an attitude towards thinking about the future, we explored whether chess players would choose the future options more often than non-chess players. Chess players’ experience in evaluating future positions and outcomes in chess may enhance their ability to make decisions that favour long-term benefits over immediate rewards. This is an exploratory research question, and it is also based in previous studies showing a positive correlation between choosing the future options and accuracy in the CRT (see Frederick 2005, but see Campitelli and Labollita 2010 for the opposite result).

Risky choice. Risky choice tasks have been used in behavioural economics and psychology of decision making (Kahneman and Tversky 1979, 1984; Tversky and Kahneman 1974), and it was used in previous studies relating these tasks to the CRT (Campitelli and Labollita 2010; Frederick 2005; Oechssler et al. 2009). As in intertemporal choice, there are no correct answers, risky choice tasks reveal preferences. Participants are given the option of choosing a sure gain or loss option (e.g., receiving or losing $10 for certain) or an uncertain gain or loss option (e.g., receiving or losing $50 with a probability of 25%). One of the options has a higher expected value (i.e., probability of the outcome × magnitude of the outcome) and according to expected utility theory (Bernoulli [1738] 1954; von Neumann and Morgenstern 1944), should be the preferred option for a rational decision maker. Given that chess playing involves calculating the outcomes of alternatives, we explored whether they would choose more often than the non-chess players the options with the highest expected value.

Summing up, given that planning and cognitive reflection are cognitive processes that occur in a chess game, we predicted that chess players would outperform non-chess players in TOL and CRT. Moreover, given that look-ahead search in chess involves a simulation of future events, we hypothesise that they would choose more future options in ITC. Regarding the risky choice tasks we predicted that chess players would prefer options with higher expected value. On the other hand we predicted that non-chess players would be loss averse; therefore, as predicted by prospect theory (Kahneman and Tversky 1979), they would avoid taking risks in the domain of gains, even when the expected value of the uncertain option is higher than that of the sure option. Furthermore, they would tend to choose uncertain options in the domain of losses, even when the uncertain option is of lower expected value than the sure option.

Importantly, the predictions for TOL and CRT are based on an analysis of the shared cognitive processes with chess; thus, they are theoretically justified. On the other hand, the predictions regarding ITC and risky choice are exploratory.

## 2. Methods

### 2.1. Participants

Twenty-five Argentine chess players (21 male; *M_age_* = 27.8, *SD* = 9.7, range = 15–55) and twenty-five non-chess players (21 male; *M_age_* = 27.0, *SD* = 6.1, range = 17–41) who were second-year undergraduate psychology students in a university in Buenos Aires participated in this study. The sample of non-chess players was collected after obtaining the sample of chess players in order to be able to match the average age and the gender distribution. The chess players sample was highly qualified (*M* international rating (Elo) = 2405, *SD* = 122), which is 4.5 standard deviations above the mean of internationally rated chess players. It contained 7 international grandmasters, 9 international masters, 5 masters of the international federation, and 4 players with international rating that were not masters. We requested the chess players to indicate the years of education (*M* = 14; *SD* = 2.36, range = 11–20). We did not request the university students the same question; however, estimating 1 year of preprimary education, 7 years of primary education, 5 years of secondary education, and 1 year in their undergraduate degree indicates that, on average, the two samples have similar number of years of education.

### 2.2. Material

The tasks we used were Tower of London (TOL; Shallice 1982); the cognitive reflection test (CRT; Frederick 2005); intertemporal choices (ITC); and risky choices (see Appendix A for all the problems presented to the participants). TOL is a standard task used to study planning abilities (see Figure 1). It contains a graphical representation of three pegs and three balls. There is an initial state and a final state. Participants have to provide the sequence of moves that lead from the initial state to the final state. We presented 5 problems with varying levels of difficulty, and we obtained the average percentage of problems solved.

The cognitive reflection test (CRT, Frederick 2005) contains three mathematical problems that typically trigger an “intuitive” but wrong option. In order to give the right answer participants have to refrain their impulse of reporting the intuitive option and engage in simple mathematical calculations (see Frederick 2005 for a detailed explanation). One of the problems is the following: “A bat and a ball cost $1.10 in total. The bat costs $1.00 more than the ball. How much does the ball cost? _____ cents”. We calculated the average number of correct problems solved.

We presented 3 ITC questions in which participants had to decide which of two options they preferred: receiving an amount of money in the present or a higher amount of money in the future (e.g., $3400 this month or $3800 next month). We calculated the average proportion of choices of the future options. In risky choices, participants were presented with situations in which they had to choose between a certain option of gaining or losing an amount of money or a chance of gaining or losing an amount of money. We grouped these questions onto three categories: situations where the expected value of the uncertain option was **h**igher than the **e**xpected **v**alue of the certain option, in the domain of **g**ains (HEVg) (e.g., $1000 for sure or a 90% chance of $5000, in which the expected value of the certain option is 1 × $1000 = $1000, and the expected value of the chance option is 0.9 × $5000 = $4500); situations where the expected value of the uncertain option was **l**ower than the **e**xpected **v**alue of the certain option, in the domain of **g**ains (LEV_g_) (e.g., winning $100 for sure (expected value: 1 × $100 = $100) or a 25% chance of winning $200 (expected value: 0.25 × $200 = $50); and situations where the expected value of the uncertain option was **l**ower than the **e**xpected **v**alue of the certain option, in the domain of **l**osses (LEV_l_) (e.g., lose $50 for sure (expected value: 1 × −$50 = −$50 or a 10% chance of losing $800 (expected value: 0.10 × −$800 = −$80)). (See Appendix B for the calculation of the expected value of each option). We calculated the proportion of uncertain choices in each category.

### 2.3. Statistical Analysis

The statistical analyses were conducted with the free statistical software JASP (JTeam 2024), which was developed to conduct Bayesian statistics with Bayes factors (see Wagenmakers et al. 2018 for an explanation of default parameters used in JASP). We tested the hypotheses comparing chess players with non-chess players using the Bayesian alternative to t-test provided by JASP, which includes the use of Bayes Factors. For all the hypothesis testing analyses with Bayes Factors, we used the default priors provided by the R package Bayesfactor (Morey et al. 2015), which is embedded in JASP. The figures were generated using the free statistical software R (RStudioTeam 2020).

The analysis consists of comparing a mathematical model of the alternative hypothesis (i.e., that there is a difference between the chess players and the non-chess players) with a mathematical model of the null hypothesis (i.e., there are no differences between chess players and non-chess players). For a full explanation of the process, we refer the reader to Wagenmakers et al. (2018); here we present a simplified explanation. Both models contain a probability distribution over possible values of the parameter δ, where δ = (population mean of group 1 − population mean of group 2)/combined population standard deviation).

The model of the null hypothesis contains a probability distribution over parameter values with a spike on δ = 0, indicating that the only possible value for δ in this model is 0; therefore, the difference between the two groups is 0. The model of the alternative hypothesis uses a Cauchy distribution with values closer to 0 being more likely and values further from zero (both positive and negative) being less likely, following the knowledge from the research in psychology that effect sizes very far from zero are extremely unlikely. When the hypothesis was directional (i.e., in CRT, TOL and ITC), the Cauchy distribution was censored at 0. When these mathematical models are combined with a statistical model of the data (i.e., the traditional statistical models such as a t distribution), each model makes a prediction of the probability of the data. For example, the probability of observing no differences or very small differences between groups has a higher probability in the model of the null hypothesis than in the model of the alternative hypothesis; on the other hand, the probability of observing moderate to large differences between groups is higher in the model of the alternative hypothesis than in the model of the null hypothesis. Once the data is observed, the model comparison is performed by calculating a Bayes factor (BF), which is the ratio between the probability of observing the data in one of the models and the probability of observing the data in the other model.

Bayes Factors are identified as BF10 or BF01. BF10 indicates we are considering the evidence for the alternative hypothesis (1) relative to that for the null hypothesis (0); BF01 refers to the evidence in favour of the null hypothesis relative to that of the alternative hypothesis. Given that BF10 is a ratio between the probability of the data under the alternative hypothesis and the probability of the data under the null hypothesis, when BF10 is greater than 1, the evidence is in favour of the alternative hypothesis and when BF10 is lower than 1, the evidence is in favour of the null hypothesis. When BF10 values are higher than 1, we interpret the results as the alternative hypothesis being the Bayes factor value times as likely as the null hypothesis. For example, if BF10 = 8, we interpret the results as the probability of the alternative hypothesis being 8 times that of the null hypothesis. When the BF10 is lower than 1, we calculate BF01 = 1/BF10 and we interpret the results in terms of the null hypothesis relative to the alternative hypothesis. For example, if BF10 = 0.20, we interpret the results as the probability of the null hypothesis being 5 times (1/0.20 = 5) that of the alternative hypothesis. We also made estimation of population means using a Bayesian posterior distribution. We report the lower bound and upper bound of the 95% credible interval of that posterior distribution.

## 3. Results

**Figure 2:**
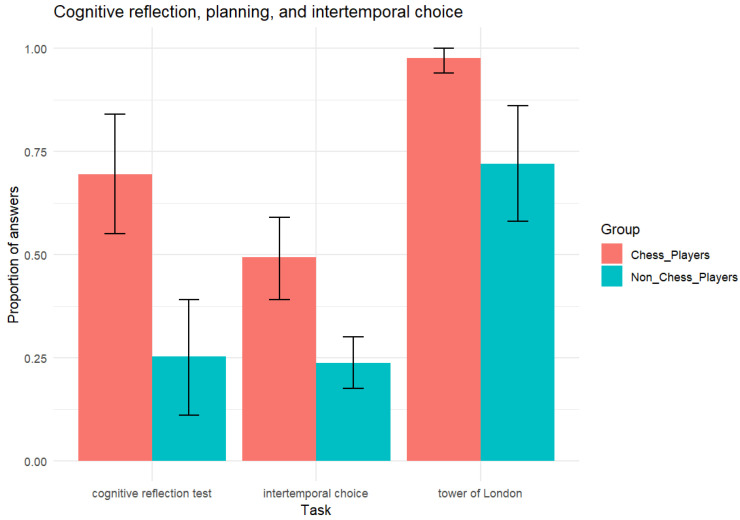
Proportion of correct answers (TOL and CRT) and future choices (ITC) as a function of expertise. Note. The error bars correspond to the 95% credible interval of the posterior distribution for the mean in each group in each task. For CRT and TOL the y-axis indicates the proportion of correct answers and for ITC it indicates the proportion of future answers.

A table with descriptive statistics, credible intervals and Bayes factors is shown in the Appendix C. Figure 2 displays the proportion of correct problems in TOL and in CRT and the proportion of future options in ITC. In TOL, the chess players performed close to 100% (*M* = .976, *SD* = .06, 95% credible interval = [.949, 1]) and, as predicted, outperformed the non-chess players (*M* = .720, *SD* = .34, [.579, 0.861]), with the BF10 = 96.47 indicating that the probability of the alternative hypothesis is 96 times that of the null hypothesis. Also, as predicted, in CRT, chess players (*M* = .694, *SD* = .34, [.551, .837]) outperformed non-chess players (*M* = .252, *SD* = .34, [.113, .392]), with the BF10 = 1114.7 indicating that the probability of the alternative hypothesis is more than 1000 times that of the null hypothesis. Finally, also as predicted, chess players (*M* = .493, *SD* = .24, [.394, .592]) chose more future options than non-chess players (*M* = .238, *SD* = .15, [.175, .300]), with the BF10 = 901.1 indicating that the probability of the alternative hypothesis is more than 900 times that of the null hypothesis.

**Figure 3:**
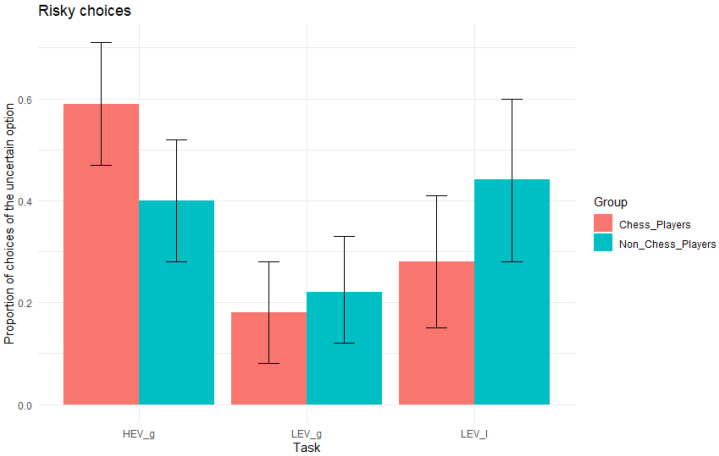
Risky choices. Proportion of uncertain choices as a function of group. Note. HEV_g_ = items where the expected value of the uncertain option is higher than that of the sure option in the domain of gains; LEV_g_ = items where the expected value of the uncertain option is lower than that of the sure option in the domain of gains; LEV_l_ = items where the expected value of the uncertain option is lower than that of the sure option in the domain of losses. The error bars correspond to the 95% credible interval of the posterior distribution for the mean in each group in each task.

Figure 3 shows that proportion of risky choices in each category as a function of group. As predicted, chess players (*M* = .590, *SD* = .28, [.471, .709]) chose more uncertain options than non-chess players (*M* = .400, *SD* = .29, [.277, .523]) in HEVg, with a BF10 = 4.54, indicating that the probability of the alternative hypothesis is 4.54 times that of the null hypothesis. Also, as predicted, chess players (*M* = .180, *SD* = .24, [.079, .281]) chose less uncertain options than non-chess players (*M*= .220, *SD* = .25, [.115, .325]) in LEVg; however, the BF10 = 0.449 indicates that the evidence is slightly in favour of the null hypothesis, the probability of which is 2.23 (i.e., 1/0.449 = 2.23) times that of the alternative hypothesis. In LEVl, as predicted, chess players (*M* = .280, *SD* = .31, [.150, .410]) chose less uncertain options than non-chess players (*M*= .441, *SD* = .38, [.283, .599]); however, the BF10 = 1.52 indicates that the evidence is only slightly in favour of the alternative hypothesis, the probability of which is only 1.52 times that of the null hypothesis.

## 4. Discussion

We hypothesized that chess players would perform better than non-chess players in a planning task (TOL), in a cognitive reflection task (CRT), and that they would have more preference for future more rewarding options than present less rewarding options (ITC) than non-chess players. Finally, we hypothesized that the proportion of choices of options with the highest expected value would be higher in chess players than in non-chess players. The results regarding the first three hypotheses are straightforward: our hypotheses were supported by the data, with the alternative hypothesis of differences between chess players and non-chess players being much more likely than that of the null hypothesis of no differences. Given that our data were publicly available (see the dataset in figshare in Campitelli and Labollita 2016) the effect sizes for TOL and CRT were used in the meta-analysis conducted by Sala et al. (2017); thus, we can compare the effect sizes of this study with that of previous studies. Of the 19 effect sizes used in that meta-analysis, the CRT effect size in this study (*Cohen’s d* = 1.54, 95%CI = [0.81, 2.27]) was the largest effect size and the TOL effect size (*Cohen’s d* = 1.23, 95%CI = [0.54, 1.92]) was the third largest effect size, and both much larger than the average effect size of the meta-analysis (*Cohen’s d* = 0.49, 95%CI = [0.26, 0.72]). 

Given that we deliberately chose CRT and TOL as candidates for large effect sizes, the result is not surprising. Slightly surprising, however, is that, unlike CRT, which was not investigated in previous studies in the context of differences between chess players and non-chess players, TOL was investigated in two studies and the effect sizes in those studies differ from the one obtained in our study: *Cohen’s d* in Unterrainer et al. (2006) = 0.72, 95%CI = [0.05, 1.39] and *Cohen’s d* in Unterrainer et al. (2011) = 0.25, 95%CI = [−0.34, 0.84]. The Unterrainer et al. (2006) study is the closest in design and results to our study and showed a significant effect size, albeit smaller than ours. The differences may be explained by the differences in the chess samples. In Unterrainer et al. (2006) the *M Elo* = 1683 (range = 1250–2100), whereas our sample is more than 3.5 standard deviations stronger—*M Elo* = 2405 (range = 2159–2607), with all the players in our sample having a higher Elo rating than those in Unterrainer et al. (2006).

Unterrainer et al. (2006) measured the time participants took to plan the answer before executing it, showing that chess players spent a lot more time than non-chess players for planning. Based on this result, they considered the possibility that the differences between chess players and non-chess players were due to being more motivated than the non-chess players, rather than possessing higher planning skills. In order to test that hypothesis, Unterrainer et al. (2011) decided to restrict the time the participants were allowed to plan. In those conditions, the effect was diminished and even though there was an advantage to chess players, the difference did not reach significance. Notice that their sample was 3 standard deviations weaker than ours—*M* Elo = 1808.6 (range = 1209–2303)—and this may explain the difference with our study, but not the difference with Unterrainer et al. (2006). Unfortunately, we did not measure the time participants spent to solve the problem, but we acknowledge that restricting the planning time could have reduced the effect size in our study. However, the look ahead search in TOL and in chess requires spending time before executing a move. Thus, restricting the time may affect the capacity of the players to apply their look ahead search to choose the correct first move. Of course, with the data available, we cannot unconfound the motivational and the skill effects, but there are good reasons to continue investigating the differences in TOL between chess players and non-chess players in future studies.

The finding that chess players are superior to non-chess players in CRT may be linked to the study of Bilalić et al. (2008b) that found that chess players were able to avoid reporting an intuitive but not efficient move in order to solve chess problems. Indeed, chess players were able to avoid the Einstellung or set effect (see Blech et al. 2020; Luchins 1942). It is important to emphasize that the CRT effect size found in the study is the largest of the effect sizes investigated relative to cognitive abilities differences between chess players and non-chess players. We cannot establish whether chess improves cognitive reflection or whether those with high cognitive reflection are attracted to play chess, but cognitive reflection should be considered a candidate target for studies aiming to show that chess playing is transferable to other abilities.

The finding that chess players chose more future options than non-chess players in ITC is intriguing. Given that our design does not allow for establishing causality, it would be interesting in future studies to investigate whether playing chess affords chess players the possibility of better evaluating future options or modifying their preferences towards future options. Chess players have practice in considering future states of a chess game. This may dispose them to consider factors that make a future larger reward more valuable. On the other hand, it may be the case that people who have preferences for waiting for future rewards are attracted to play chess, a game that requires patience and investing time (both before games and during games) and effort to achieve a reward.

The fourth hypothesis, that chess players would choose more often the options that have the highest expected value than non-chess players was partially supported, and the support is weak. Nonetheless, although chess players were far from behaving entirely guided by expected value (i.e., 100% of uncertain choices in HEV_g_ and 0% of uncertain choices in LEV_g_ and in LEV_l_) they were closer to those values than non-chess players.

A potential limitation of this study is that, although we controlled for age and education, we did not control for general intelligence. It is important to emphasise, however, that controlling for intelligence is not necessarily correct. According to causal graphs theory (see Pearl 2009; Pearl and Mackenzie 2018 for an explanation of causal graphs and when it is appropriate to control for variables) only when variations in intelligence cause variations in, say, planning, is it appropriate to correct for intelligence. On the other hand, if the causal relationship is the opposite (variations in planning cause variations in intelligence) controlling for intelligence would be inappropriate. It is beyond the scope of this study to discuss what the causal relationship between the variables measured in this study and intelligence is. Another limitation is that our sample size of 50 participants is relatively small, and a larger sample size would provide a more precise estimation of population parameters.

Summing up, this study found the largest and the third largest effect size in the literature investigating differences in cognitive abilities between chess players and non-chess players. Chess players were superior in cognitive reflection and planning. Moreover, chess players have shown preferences for future more rewarding options than present less rewarding options, with a very large effect size as well. Given our study does not allow us to establish whether chess playing causes the behaviour observed in the measured variables or the differences between chess players and non-chess players existed before chess players started playing chess, future studies should investigate the causal relationship between chess playing and planning, cognitive reflection, risky choice, and intertemporal choice. The results of this study can inform researchers who are interested in investigating the transfer of knowledge and skill from chess to other areas, with cognitive reflection and planning being excellent targets for those potential studies.

## Data Availability

The dataset is publicly available in figshare (https://figshare.com/articles/dataset/Campitelli_Labollita_Datset_Chessplayers_Students/3172813?file=4941574 (accessed on 12 March 2025)).

## Appendix A

*Tower of London*

This game contains three pegs and three balls with a hole in the middle such that the balls can slide from top to bottom of the peg. In all the problems, there is an initial state (left panel) and an end state (right panel). The goal is to transform the initial state onto the end state by moving the balls from peg to peg, following these rules:

1. You can move one ball at a time.
2. A ball cannot move when there is another ball on top of it.
3. There can be a maximum of three balls in the left peg, a maximum of two balls in the middle peg, and a maximum of one ball in the right peg.

There are five problems, and in each of them the number of moves required to solve the problem is indicated. You should first think what the sequence of correct movements is to go from the initial state to the end state. Once you consider that you have the correct answer, write down the sequence below the problem, following these rules:

- The black ball is referred to as B; the grey ball is referred to as G; and the white ball is referred to as W.
- The left peg is referred to as 1; the middle peg is referred to as 2, and the right peg is referred to as 3.
- Write down each movement indicating the name of the ball that moves and then the number of the peg to which the ball will move.

*Problem 1. Number of Moves for the Solution: 4*

*Problem 2. Number of Moves for the Solution: 4*

*Problem 3. Number of Moves for the Solution: 5*

*Problem 4. Number of Moves for the Solution: 6*

*Problem 5. Number of Moves for the Solution: 7*

*Cognitive Reflection Test* (Frederick 2005)

Here there are three problems. Complete the blank space with the correct answer.

(1) A bat and a ball cost $1.10 in total. The bat costs $1.00 more than the ball. How much does the ball cost? _____ cents
(2) If it takes 5 machines 5 min to make 5 widgets, how long would it take 100 machines to make 100 widgets? _____ minutes
(3) In a lake, there is a patch of lily pads. Every day, the patch doubles in size. If it takes 48 days for the patch to cover the entire lake, how long would it take for the patch to cover half of the lake? _____ days

*Intertemporal Choice*

In the following questions, decide which of the options you prefer. None of the options is better than the other; we only want to know what your preference is.

(1) (a) Receive $3400 this month, or (b) receive $3800 the following month.
(2) (a) Receive $100 now, or (b) receive $140 in a year.
(3) (a) Receive $100 now, or (b) receive $1100 in 10 years.

*Risky Choice*

In the following questions, decide which of the options you prefer. None of the options is better than the other; we only want to know what your preference is.

(HEV_g_)

(4) (a) Receive $1000 for certain or (b) Having a 90% chance of receiving $5000.
(5) (a) Receive $100 for certain or (b) Having a 75% chance of receiving $200.
(6) (a) Receive $100 for certain or (b) Having a 75% chance of receiving $150.
(7) (a) Receive $500 for certain or (b) Having a 15% chance of receiving $1,000,000

(LEV_g_)

(8) (a) Receive $100 for certain or (b) Having a 25% chance of receiving $200.
(9) (a) Receive $5 for certain or (b) Having a 4% chance of receiving $80.

(LEV_l_)

(10) (a) Lose $100 for certain or (b) Having a 75% chance of losing $200.
(11) (a) Lose $50 for certain or (b) Having a 10% chance of losing $800.
(12) (a) Lose $100 for certain or (b) Having a 3% chance of losing $7000.

## Appendix B. Calculation of Expected Value in Risky Choice, with the Option with Highest Expected Value in Bold

(HEV_g_)

(4) (a) 1 × $1000 = $1000 or (b) .90 × $5000 = $4500.
(5) (a) 1 × $100 = $100 or (b) .75 × $200 = $150
(6) (a) 1 × $100 = $100 or (b) .75 × $150 = $112.5.
(7) (a) 1 × $500 = $500 or (b) .15 × $1,000,000 = $150,000

(LEV_g_)

(8) (a) 1 × $100 = $100 or (b) .25 × $200 = $50.
(9) (a) 1 × $5 = $5 or (b) .04 × $80 = $3.2.

(LEV_l_)

(10) (a) 1 × −$100 = −$100 or (b) .75 × −$200 = −$150.
(11) (a) 1 × −$50 = −$50 or (b) .10 × −$800 = −$80.
(12) (a) 1 × −$100 = −$100 or (b) .03 × −$7000 = −$210.

## Appendix C

**Table A1:** Descriptive Statistics, 95% Credible Intervals, and Bayes Factors.

	Chess Players				Non-Chess Players				
	*M*	*SD*	95% CI (LB)	95% CI (UB)	*M*	*SD*	95% CI (LB)	95% CI (UB)	BF10
Age	27.8	9.704			27	6.069			0.297
TOL	0.976	0.066	0.949	1.003	0.72	0.342	0.579	0.861	96.474
CRT	0.694	0.346	0.551	0.837	0.252	0.337	0.113	0.392	1114
ITC	0.493	0.24	0.394	0.592	0.238	0.151	0.175	0.3	901.1
HEV_g_	0.590	0.288	0.471	0.709	0.400	0.298	0.277	0.523	2.318
LEV_g_	0.180	0.245	0.079	0.281	0.220	0.253	0.115	0.325	0.323
LEV_l_	0.280	0.315	0.15	0.41	0.441	0.382	0.283	0.599	0.822

Note. M = Mean, SD = Standard deviation; 95% CI (LB) and (UB) refer to the lower bound and upper bound of the 95% credible interval of the posterior distribution of the mean, respectively. See the text for other abbreviations. The number of participants was 25 in each group.
